# TGR5 Regulates Macrophage Inflammation in Nonalcoholic Steatohepatitis by Modulating NLRP3 Inflammasome Activation

**DOI:** 10.3389/fimmu.2020.609060

**Published:** 2021-02-22

**Authors:** Yong Shi, Wantong Su, Lei Zhang, Chengyu Shi, Jinren Zhou, Peng Wang, Hao Wang, Xiaoli Shi, Song Wei, Qi Wang, Johan Auwerx, Kristina Schoonjans, Yue Yu, Rui Pan, Haoming Zhou, Ling Lu

**Affiliations:** ^1^ Jiangsu Key Laboratory of Molecular and Translational Cancer Research, The Affiliated Cancer Hospital (Jiangsu Cancer Hospital), Nanjing Medical University, Nanjing, China; ^2^ Hepatobiliary Center, The First Affiliated Hospital of Nanjing Medical University & Research Unit of Liver Transplantation and Transplant Immunology, Chinese Academy of Medical Sciences, Nanjing, China; ^3^ State Key Laboratory of Reproductive Medicine, Nanjing Medical University, Nanjing, China; ^4^ Jiangsu Collaborative Innovation Center of Biomedical Functional Materials, College of Chemistry and Materials Science, Nanjing Normal University, Nanjing, China; ^5^ Jiangsu Key Lab of Cancer Biomarkers, Prevention and Treatment, Collaborative Innovation Center for Personalized Cancer Medicine, Nanjing Medical University, Nanjing, China; ^6^ Metabolic Signaling, School of Life Sciences, Institute of Bioengineering, Ecole Polytechnique Federale de Lausanne, Lausanne, Switzerland

**Keywords:** nonalcoholic steatohepatitis, G protein-coupled bile acid receptor 1, macrophages, inflammation, NLRP3 inflammasome

## Abstract

Nonalcoholic steatohepatitis (NASH) is a chronic liver disease associated with dysregulation of liver metabolism and inflammation. G-protein coupled bile acid receptor 1 (TGR5) is a cell surface receptor that is involved in multiple metabolic pathways. However, the functions of TGR5 in regulating macrophage innate immune activation in NASH remain unclear. Here, we found that TGR5 expression was decreased in liver tissues from humans and mice with NASH. Compared to wild type (WT) mice, TGR5-knockout (TGR5^−/−^) mice exhibited exacerbated liver damage, increased levels of proinflammatory factors, and enhanced M1 macrophage polarization. Moreover, TGR5 deficiency facilitated M1 macrophage polarization by promoting NLRP3 inflammasome activation and caspase-1 cleavage. Taken together, our findings revealed that TGR5 signaling attenuated liver steatosis and inflammation and inhibited NLRP3-mediated M1 macrophage polarization in NASH.

## Introduction

Nonalcoholic fatty liver disease (NAFLD) is a clinicopathological syndrome characterized by excessive intrahepatic fat deposition (more than 5%) caused by factors in addition to alcohol and other definitive liver-damaging factors (1). As a complex multifactorial metabolic disease, NAFLD is associated with a variety of factors, including insulin resistance, obesity, and genetic susceptibility. NAFLD is classified as simple fatty liver (SFL), nonalcoholic steatohepatitis (NASH), and cirrhosis-related based on disease progression (2). Recent studies have shown that NASH is a more serious process in the development of NAFLD, characterized by lobular inflammation and pericellular fibrosis, which is likely to develop into cirrhosis and hepatocellular carcinoma (3). NASH is an inflammatory disease with complex pathophysiological processes, involving the recruitment of immune cells and the activation of proinflammatory signaling, in which macrophages are key (4). Lipid toxicity and hepatocyte injury induce the activation of macrophages, leading to monocyte infiltration and proinflammatory cytokine production, which are involved in the development of NASH (5).

G protein-coupled bile acid receptor 1 (TGR5, also known as GPBAR1) is a cell surface receptor that specifically binds to bile acids (6, 7). In addition to conjugated bile acids and unconjugated bile acids, various steroids can act on TGR5 receptors to activate a variety of intracellular signaling pathways (8, 9). Previously, some studies have demonstrated that TGR5 is primarily expressed in macrophages (also known as Kupffer cells, KCs) in the liver (10, 11). The expression of TGR5 in macrophages is mainly involved in anti-inflammation and anti-apoptosis, which has an important influence on the occurrence and development of multiple liver diseases (12). In LPS-induced damage, TGR5 inhibits the release of cytokines from macrophages, preventing the overproduction of cytokines and liver damage (13). TGR5 knockout mice with an overload of bile acids induced by cholic acid feeding or common bile duct ligation, exhibited increased macrophage infiltration and severe cellular necrosis. It is speculated that activation of TGR5-dependent signaling pathways may lead to a decrease in macrophage phagocytic activity (14).

Macrophages can be divided into proinflammatory macrophage phenotype M1 and anti-inflammatory macrophage phenotype M2 based on their functions, secreted cytokines and transcriptional profiles (15). The inflammatory response can regulate M1/M2 polarization of macrophages, and the released proinflammatory cytokines, such as tumor necrosis factor-α (TNF-α) and anti-inflammatory cytokine interleukin-10 (IL-10) maintain homeostasis. Previous research has shown that hyperglycemia regulates the polarization of KCs in acetaminophen-induced acute liver injury *via* AMPK/PI3K/AKT-mediated oxidative stress (16). While there are only a few studies on TGR5 regulating M1/M2 polarization. In inflammatory bowel diseases, TGR5 gene ablation enhances the recruitment of macrophages, and the expression of IL-10 and TGF-β in mice stimulated by Bar501 (a TGR5 agonist) is increased (17).

As a member of the nucleotide-binding domain leucine-rich repeat (NLR) family, the NLRP3 (NLR and pyrin domain containing receptor 3) inflammasome has been shown to play a role in immunity and human disease (18). Studies have shown that activation of the NLRP3 inflammasome in macrophages is induced by TNF-α through activation of NF-κB (19). In addition, NLRP3 is associated with insulin resistance and the development of NASH. Excessive accumulation of lipids in dead hepatocytes activates macrophages with high levels of NLRP3 and caspase-1 (20).

However, the detailed mechanism underlying the beneficial aspect of TGR5 in NASH remains unknown. In our study, we investigated how TGR5 affects M1/M2 polarization of macrophages. Overall, our results demonstrated that TGR5 signaling attenuates macrophage-related inflammation and inhibits M1 polarization of macrophages through NLRP3 signaling in NASH.

## Materials and Methods

### Human Liver Samples

Liver samples were obtained from patients with or without fatty liver (6 samples in each group, **Table S1**) at the First Affiliated Hospital of Nanjing Medical University, all of whom had signed informed consent forms. The use of human tissue was approved by the Ethics Committee of the Affiliated Hospital of Nanjing Medical University (approval number 2019-SR-127).

### Animals

Wild type (WT) male C57BL/6J mice (8 weeks old, 15–30 g) and TGR5 knockout (TGR5^-/-^) male mice (a gift from Kristina Schoonjans, 8 weeks old, 15–30 g) were housed at the Gempharmatech Co., Ltd. (Nanjing, China) (21). Mice were fed a normal chow diet (NCD) or a methionine and choline-deficient (MCD) diet (**Table S2**) for six weeks and were divided into four groups (WT-NCD, TGR5^-/-^–NCD, WT-MCD, TGR5^-/-^–MCD, n = 5/group). Mice were given sterilized water and food ad libitum. Mice were randomly divided into groups. All animal experiments were approved by the Institutional Animal Care and Use Committee of the Nanjing Medical University (approval number IACUC-1905029). Mice received humane care and were euthanized according to the Institutional Animal Care and Use Committee of Nanjing Medical University.

### Liver Function Assay

Blood samples were obtained from mice and were separated by centrifugation (3000 rpm for 10 minutes). Then, the alanine transaminase (ALT) and aspartate transaminase (AST) levels in the serum were determined using a biochemical analyzer (Olympus, Tokyo, Japan). Triglyceride content was measured using the Triglyceride Reagent Set (Pointe Scientific, Canton, MI).

### Western Blotting

Cellular and liver tissue proteins were extracted using RIPA lysis buffer (Beyotime Biotechnology Co., Ltd., China). The buffer was placed on ice for 30 minutes and vortexed every 10 minutes. Then, the solution was centrifuged at 12000 rpm for 10 minutes at 4℃. Approximately 25 mg protein were separated by SDS-PAGE and then transferred onto nitrocellulose membranes (Sunshine Biotechnology, China). After blocking membranes with skim milk powder at 25℃ for 2 h, membranes were incubated with antibodies overnight. Antibodies against β-actin, GAPDH, NLRP3, IL-1β, caspase-1, cleave-caspase-1, iNOS, STAT-1, STAT-6, p-STAT1, p-STAT6 (#4970, #5174, #15101, #31202, #24232, #89332, #13120, #14994, #5397, #9177, #56554, Cell Signaling Technology, Danvers, MA, USA), and TGR5, and CD206 (#ab72608, #ab64693, Abcam, Cambridge, MA, USA) were diluted 1:1000 in a primary antibody buffer (Beyotime Biotechnology Co., Ltd., China). After washing 3 times with TBST buffer, membranes were subsequently incubated with HRP-conjugated goat anti-rabbit IgG secondary antibody (Dilution ratio: 1:2000, Santa Cruz Biotechnology, Santa Cruz, CA) at room temperature for 2 h. Images were acquired using darkroom development techniques for chemiluminescence (WBKL0100; Millipore Sigma) and were analyzed with Image J software (National Institutes of Health, USA).

### ELISA

Following the manufacturer’s protocol, cytokine levels of tumor necrosis factor-α (TNF-α), interleukin-6 (IL-6), IL-1β, IL-4, IL-10, and IL-18 in the serum and supernatant were analyzed using ELISA kits (R&D System, Shanghai, China).

### Cell Isolation and Culture

Bone marrow cells were isolated from the femurs and tibias of WT and TGR5^-/-^ mice (22). Then, cells (5*10^5/ml) were transferred into 8 petri dishes (100 mm) and cultured in 10 ml DMEM supplemented with 10% fetal calf serum and M-CSF (20 ng/ml; Sino Biologic, Beijing, China) at 37℃ and 5% CO2. After 6 to 8 h, adherent cells were collected. The culture medium was renewed every 3 days. Cell growth was monitored periodically with an inverted microscope (Olympus, Tokyo, Japan). After 7 days, bone marrow cells had differentiated into bone marrow-derived macrophages (BMDMs), and the cell counting kit-8 was performed to assess cell viability (Beyotime Biotechnology Co., Ltd., China). BMDMs were then stimulated using palmitic acid (PA, 200 mM, Sigma, Saint Louis, MO, USA) (23) or CY-09 (10 µM, Cell Signaling Technology, Danvers, MA, USA) (24) for 24 h. Then, cells and supernatants were used in subsequent experiments. All experiments were performed in triplicate.

### Histological Analysis

Hematoxylin & eosin (H&E, original magnification, 200X and 400X, Staining Kit, Beyotime Biotechnology Co., Ltd., China) and Sirius Red (original magnification, 200X, Staining Kit, Beyotime Biotechnology Co., Ltd., China) staining were performed on liver tissue sections. Inflammation and tissue damage were observed by light microscopy. Liver macrophages were detected using rat anti-mouse F4/80 monoclonal antibody (BD Biosciences, San Jose, CA, USA). F4/80 positive cells were counted on high-power (200X) fields per slide. Immunofluorescence staining was used to assess NLRP3 inflammasome activation and macrophages polarization. Liver samples from mice were incubated with antibodies, including NLRP3, caspase-1, ASC, CD68, iNOS, and CD206 (Cell Signaling Technology, Danvers, MA, USA; Abcam, Cambridge, MA, USA). Images were obtained using an inverted microscope (Olympus, Tokyo, Japan) and were analyzed using Image J software (National Institutes of Health, USA). A diagnosis of NAFLD was determined according to the NAFLD activity score, and the score was determined according to the published criteria (25), which was determined by steatosis, inflammation, and balloon swelling.

### Real-Time Quantitative PCR

Following the protocol, total ribonucleic acid (RNA) in liver tissues and cells was extracted using the RNA Extraction kit (Invitrogen, California, USA). Then, cDNA was obtained by reverse transcription using a reverse transcriptase kit (Takara Bio, Shiga, Japan). A real-time quantitative polymerase chain reaction (PCR) was then performed. Expression of target genes was normalized by GAPDH. All experiments were performed in triplicate. Primers are shown in **Table 1**.

**Table 1:** List of primer used for RT-PCR.

Gene	Forward primer	Reverse primer
GAPDH	AGGTCGGTGTGAACGGATTTG	GGGGTCGTTGATGGCAACA
TNF-α	CAGGCGGTGCCTATGTCTC	CGATCACCCCGAAGTTCAGTAG
IL-6	CTGCAAGAGACTTCCATCCAG	AGTGGTATAGACAGGTCTGTTGG
IL-4	CCCCAGCTAGTTGTCATCCTG	CAAGTGATTTTTGTCGCATCCG
IL-10	GCTGGACAACATACTGCTAACC	ATTTCCGATAAGGCTTGGCAA
TGR5	TGCTTCTTCCTAAGCCTACTAC	CTGATGGTTCCGGCTCCATAG
CD68	TGTCTGATCTTGCTAGGACCG	GAGAGTAACGGCCTTTTTGTGA
CD86	TCAATGGGACTGCATATCTGCC	GCCAAAATACTACCAGCTCACT
iNOS	ACATCGACCCGTCCACAGTAT	CAGAGGGGTAGGCTTGTCTC
CD163	GGTGGACACAGAATGGTTCTTC	CCAGGAGCGTTAGTGACAGC
Arg-1	CTCCAAGCCAAAGTCCTTAGAG	GGAGCTGTCATTAGGGACATCA
CD206	CTCTGTTCAGCTATTGGACGC	TGGCACTCCCAAACATAATTTGA
IL-1β	GAAATGCCACCTTTTGACAGTG	TGGATGCTCTCATCAGGACAG
IL-18	GTGAACCCCAGACCAGACTG	CCTGGAACACGTTTCTGAAAGA
NLRP3	ATCAACAGGCGAGACCTCTG	GTCCTCCTGGCATACCATAGA

### Statistical Analysis

Statistical analysis was performed using GraphPad Prism 7. For all experiments where comparisons were made for more than two populations, one-way ANOVA was used. When comparisons were made between two populations, statistical significance was assessed using unpaired Student’s t-tests. All experiments were repeated three times. P-values less than 0.05 (P < 0.05) were accepted as statistically significant.

## Results

### TGR5 Deficiency Exacerbates NASH

**Figure 1:**
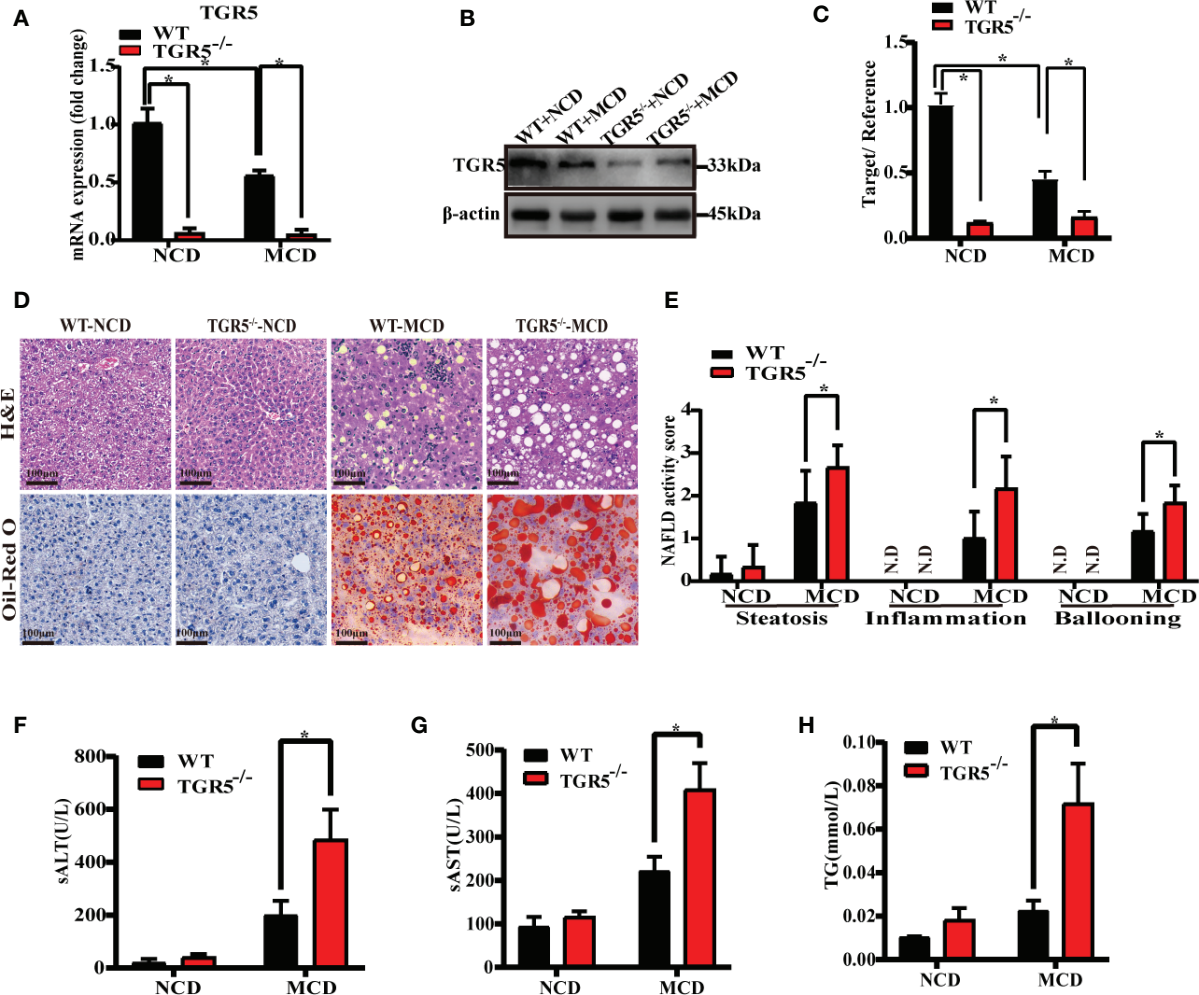
TGR5 deficiency exacerbates steatohepatitis. WT and TGR5^-/-^ mice were fed a normal or MCD diet for 6 weeks (n = 5/group). **(A)** Hepatic mRNA levels were examined by real-time quantitative PCR. **(B**, **C)** Protein expressions were examined by western blotting and relative density ratio of protein levels of TGR5 was analyzed (TGR5/β-actin). **(D)** Representative images of H&E and Oil-Red O in liver sections from mice (scale bar, 100 μm). **(E)** NAFLD activity scores were calculated, and steatosis, inflammation, and ballooning were increased in TGR5^-/-^–MCD mice. N.D., not detected. **(F**–**H)** Levels of serum ALT, AST, and hepatic triglycerides were increased in TGR5^-/-^–MCD mice. Data represent mean ± SD; *P < 0.05.

To examine the correlation between NASH and TGR5, WT and TGR5^-/-^ mice were fed NCD or MCD diet for 6 weeks. Consistent with results of the human liver, the WT-MCD mice exhibited lower protein and mRNA levels of TGR5 compared to WT-NCD mice (**Figures 1A–C**). When fed a NCD diet, WT and TGR5^-/-^ mice showed no significant steatosis or inflammation. When fed an MCD diet, both groups of mice exhibited significant steatosis, demonstrating that TGR5^-/-^ mice presented more severe fat deposition and inflammation than WT mice (**Figure 1D**). In particular, the NAFLD activity scores of TGR5^-/-^–MCD mice were strikingly higher than WT-MCD mice (**Figure 1E**). Furthermore, steatosis and liver damage in TGR5^-/-^–MCD mice were more severe, exhibiting higher levels of ALT, AST and liver triglycerides (**Figures 1F–H**). These results indicate that TGR5 may attenuate steatosis and liver injury in NASH.

### TGR5 Inhibits Macrophage-Related Inflammation in NASH

**Figure 2:**
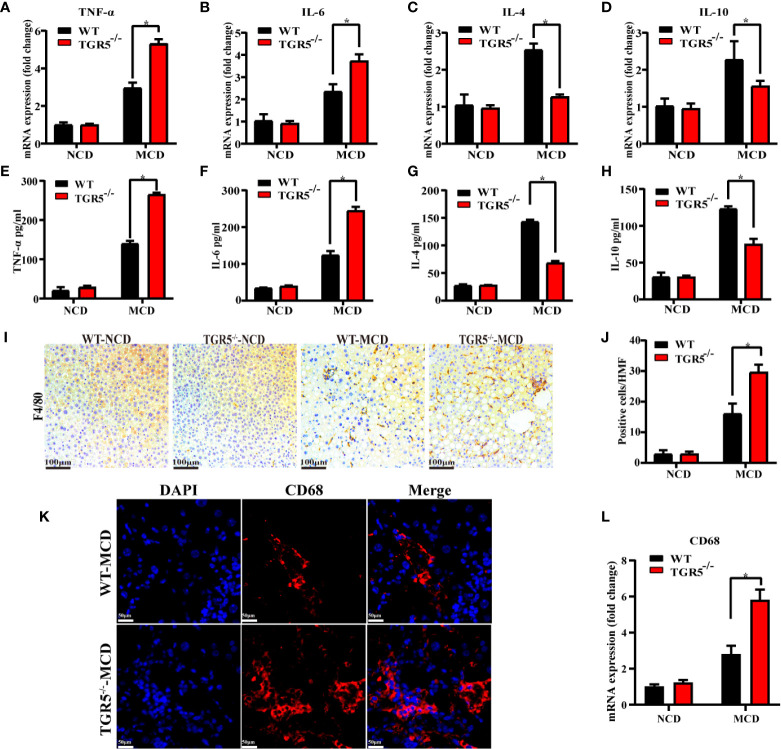
TGR5 deficiency increases inflammation and promotes macrophage accumulation in mice with NASH. **(A**–**D)** Hepatic mRNA levels of TNF-α, IL-6, IL-4, and IL-10 were measured by real-time quantitative PCR. **(E**–**H)** Serum levels of TNF-α, IL-6, IL-4, and IL-10 were measured by ELISA in mice. **(I**, **J)** Macrophage infiltration was assessed by F4/80 immunostaining (scale bar, 100 μm). Positive cells per high magnification field (HMF) were counted using Image J in 8 fields per section (n = 5). **(K)** Representative immunofluorescence images of liver samples were stained with CD68 antibodies (scale bar, 50 μm). **(L)** Hepatic mRNA levels of CD68 were measured by real-time quantitative PCR (n = 5/group). Data represent mean ± SD; *P < 0.05.

Next, intrahepatic inflammation was compared in different groups. Compared to WT-MCD mice, TGR5^-/-^–MCD mice expressed higher levels of proinflammatory cytokines TNF-α and IL-6 but lower expression of anti-inflammatory cytokines IL-4 and IL-10 (**Figures 2A–D**). Serum ELISA was subsequently performed, and the results were consistent with those reported above (**Figures 2E–H**). To evaluate whether macrophages are involved in the intrahepatic inflammation regulated by TGR5 in NASH, the number of macrophages in livers of WT and TGR5^-/-^ mice were compared (**Figures 2I, J**). As shown in **Figures 2K, L**, compared to WT mice, TGR5^-/-^ mice showed enhanced staining of CD68 and higher levels of CD68 mRNA in liver tissues post MCD diet. Collectively, the data indicated that deficiency of TGR5 in NASH increased macrophage-related inflammation.

### TGR5 Regulates M1/M2 Macrophage Polarization

**Figure 3:**
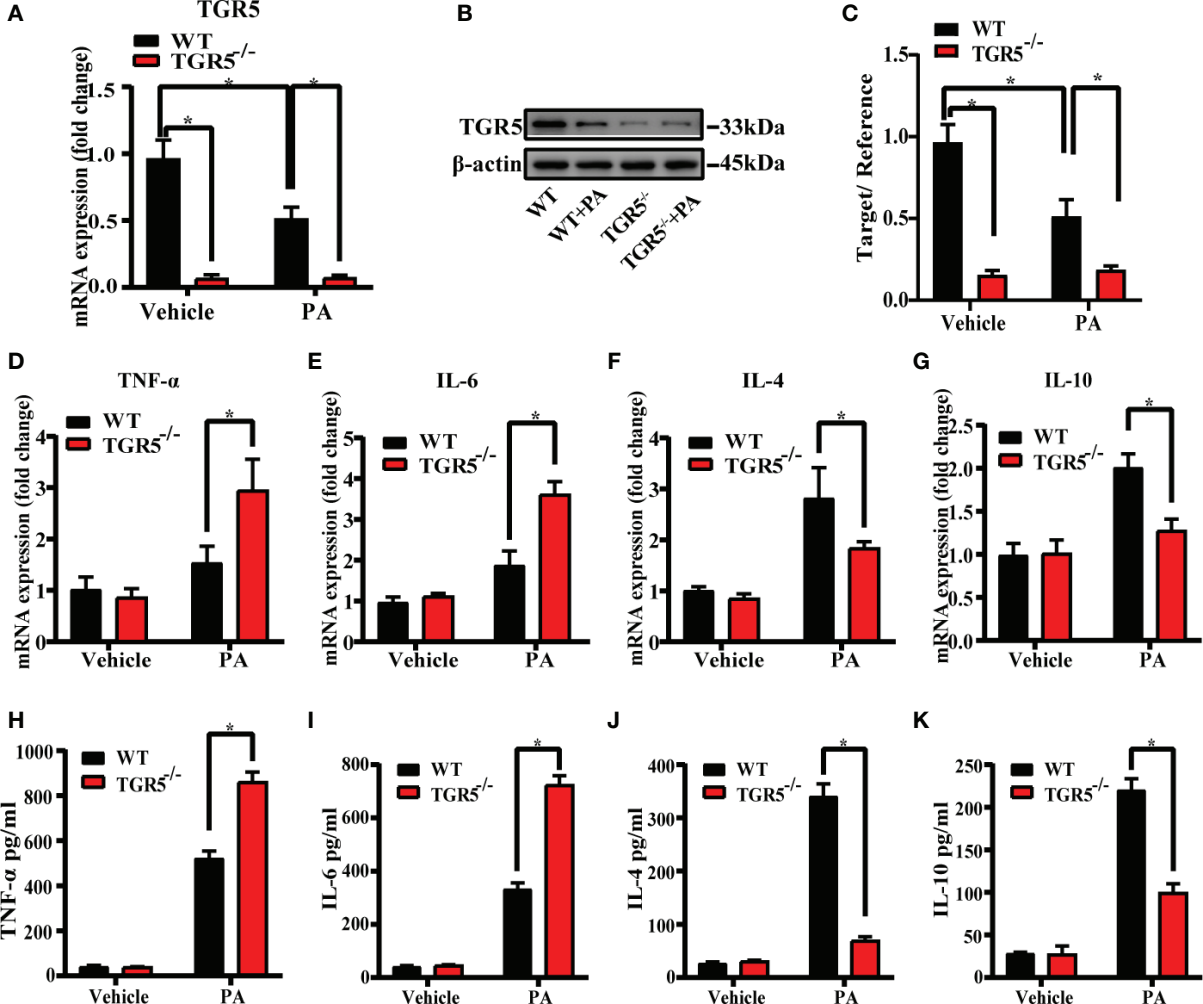
Deficiency of TGR5 in BMDMs increases inflammation under PA stimulation. **(A**, **B)** mRNA levels and protein expressions of TGR5 detected by real-time quantitative PCR and western blotting, respectively (n = 3/group). **(C)** The relative quantities of TGR5 protein to β-actin. **(D–G)** mRNA levels of TNF-α, IL-6, IL-4, and IL-10 in BMDMs determined by real-time quantitative PCR. **(H–K)** Levels of TNF-α, IL-6, IL-4, and IL-10 were measured by ELISA in cell culture supernatants. Data represent mean ± SD; *P < 0.05.

To explore the role of TGR5 in regulating the M1/M2 macrophage polarization, BMDMs from WT mice and TGR5^-/-^ mice were stimulated by PBS or PA for 24 h, respectively. PA stimulation inhibited TGR5 expression in WT BMDMs (**Figures 3A–C**). TGR5^-/-^ BMDMs exhibited higher levels of proinflammatory TNF-α and IL-6 but lower levels of anti-inflammatory IL-4 and IL-10 gene induction post-PA stimulation compared to WT BMDMs (**Figures 3D–G**). In parallel, BMDMs from TGR5^-/-^ mice under PA treatment secreted higher levels of TNF-α and IL-6 and lower levels of IL-4 and IL-10 compared to BMDMs isolated from WT mice (**Figures 3H–K**).

**Figure 4:**
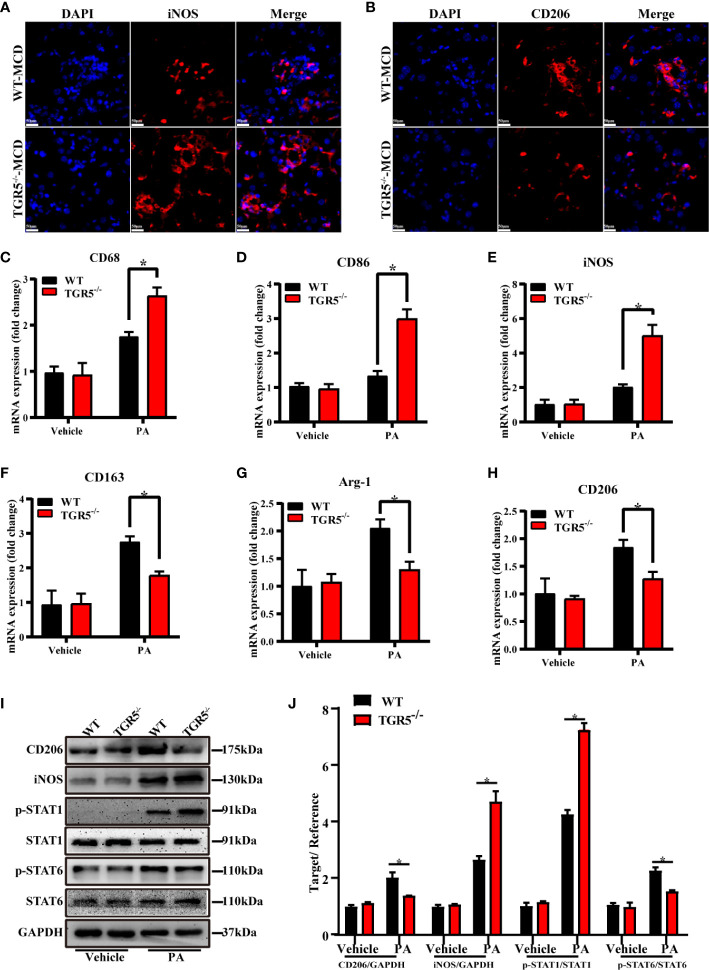
TGR5 deficiency in NASH mediates macrophages polarization in response to PA treatment. **(A, B)** Representative immunofluorescence images of liver samples were stained with iNOS and CD206 antibodies (scale bar, 50 μm). **(C–H)** The mRNA levels of M1 markers (CD68, CD86 and iNOS) and M2 markers (CD163, Arg-1 and CD206) were analyzed by real-time qPCR (n = 3/group). **(I)** CD206, iNOS, p-STAT1, STAT1, p-STAT6, STAT6 and GAPDH protein levels were detected by western blotting. **(J)** The relative quantities of CD206, iNOS protein to GAPDH, p-STAT1 protein to STAT1, p-STAT6 protein to STAT6. Data represent mean ± SD; *P < 0.05.

M1/M2 macrophage markers were next compared in different groups. Indeed, compared with WT-MCD mice, increased iNOS expression but decreased CD206 expression was found in TGR5^-/-^–MCD mice (**Figures 4A, B**). Results showed that post-PA stimulation, mRNA levels of M1 markers CD68, CD86 and iNOS in TGR5^-/-^ BMDMs were higher, while M2 markers of CD163, Arg-1, and CD206 were lower compared to WT BMDMs (**Figures 4C–H**). Furthermore, TGR5^-/-^ BMDMs were marked by increased activation of STAT1 but decreased STAT6 activation post-PA treatment (**Figures 4I, J**). These results suggest that TGR5 promotes M2 macrophage polarization.

### TGR5 Promotes M2 Macrophage Polarization by Inhibiting Activation of the NLRP3 Inflammasome

**Figure 5:**
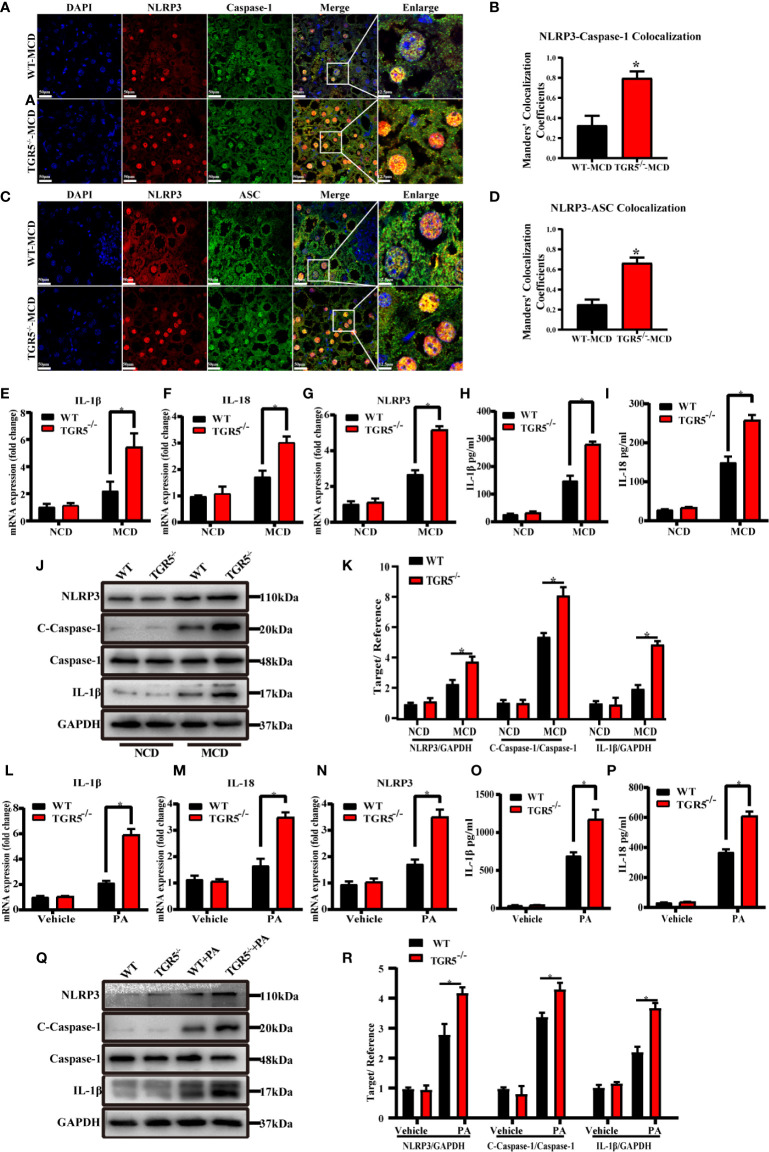
Deficiency of TGR5 actives NLRP3 inflammasome both in mice and BMDMs with NASH. **(A–D)** Representative immunofluorescence images of liver samples were stained with NLRP3, caspase-1 and ASC antibodies (scale bar, 50 μm, 12.5 μm). Colocalization of NLRP3-Caspase-1 and NLRP3-ASC were analyzed by Image J. **(E–G)** mRNA levels of IL-1β, IL-18 and NLRP3 were measured by real-time qPCR in mice (n = 5/group). **(H, I)** Production of IL-1β, IL-18 was measured by ELISA in serum. **(J)** Protein levels of NLRP3, c-caspase-1, caspase-1, IL-1β and GAPDH were examined by western blotting in mice (n = 5/group). **(K)** Western blots analysis and relative density ratio of hepatic protein levels of NLRP3, c-caspase-1 and IL-1β. **(L–N)** mRNA levels of IL-1β, IL-18 and NLRP3 were measured by real-time qPCR in BMDMs (n = 3/group). **(O, P)** Production of IL-1β, IL-18 was measured by ELISA in cell culture supernatants. **(Q)** Protein levels of NLRP3, c-caspase-1, caspase-1, IL-1β and GAPDH were detected by western blotting in BMDMs. **(R)** Western blots analysis and relative density ratio of cellular protein levels of NLRP3, c-caspase-1 and IL-1β. Data represent mean ± SD; *P < 0.05.

Although the NLRP3 inflammasome has been demonstrated to be associated with many diseases, little is known about its role in macrophages (26, 27). As shown in **Figures 5A–D**, compared to WT mice, TGR5^-/-^ mice showed enhanced staining of NLRP3, caspase-1 and ASC and much more co-localization of NLRP3-caspase-1 and NLRP3-ASC in liver tissues post MCD diet. NLRP3 mRNA and protein expression were remarkably increased in liver tissues from TGR5^-/-^–MCD mice. Compared to WT-MCD mice, expression of caspase-1, IL-1β and IL-18 were also increased in TGR5^-/-^–MCD mice (**Figures 5E–K**). Moreover, under stimulation of PA, TGR5^-/-^ BMDMs expressed higher levels of NLRP3, caspase-1, IL-1β and IL-18 than WT BMDMs (**Figures 5L–R**).

**Figure 6:**
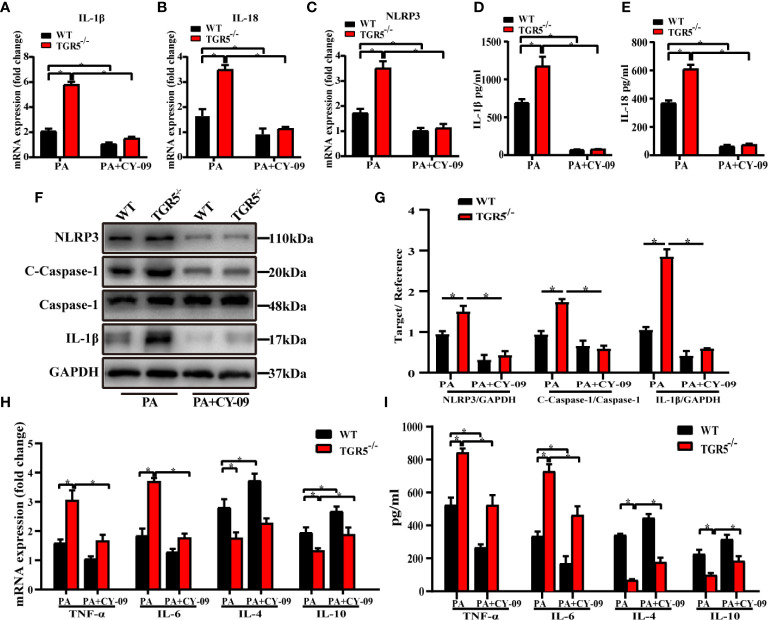
Inhibition of the NLRP3 inflammasome reduces inflammation in BMDMs. **(A–C)** mRNA levels of IL-1β, IL-18 and NLRP3 were measured by real-time qPCR in BMDMs (n = 3/group). **(D, E)** Production of IL-1β and IL-18 was measured by ELISA in cell culture supernatants. **(F)** Protein levels of NLRP3, c-caspase-1, caspase-1, IL-1β and GAPDH were detected by western blotting in BMDMs. **(G)** Western blots analysis and relative density ratio of cellular protein levels of NLRP3, c-caspase-1 and IL-1β. **(H, I)** mRNA levels and production of TNF-α, IL-6, IL-4, and IL-10 were measured by real-time quantitative PCR in BMDMs and ELISA in cell culture supernatants, respectively. Data represent mean ± SD; *P < 0.05.

**Figure 7:**
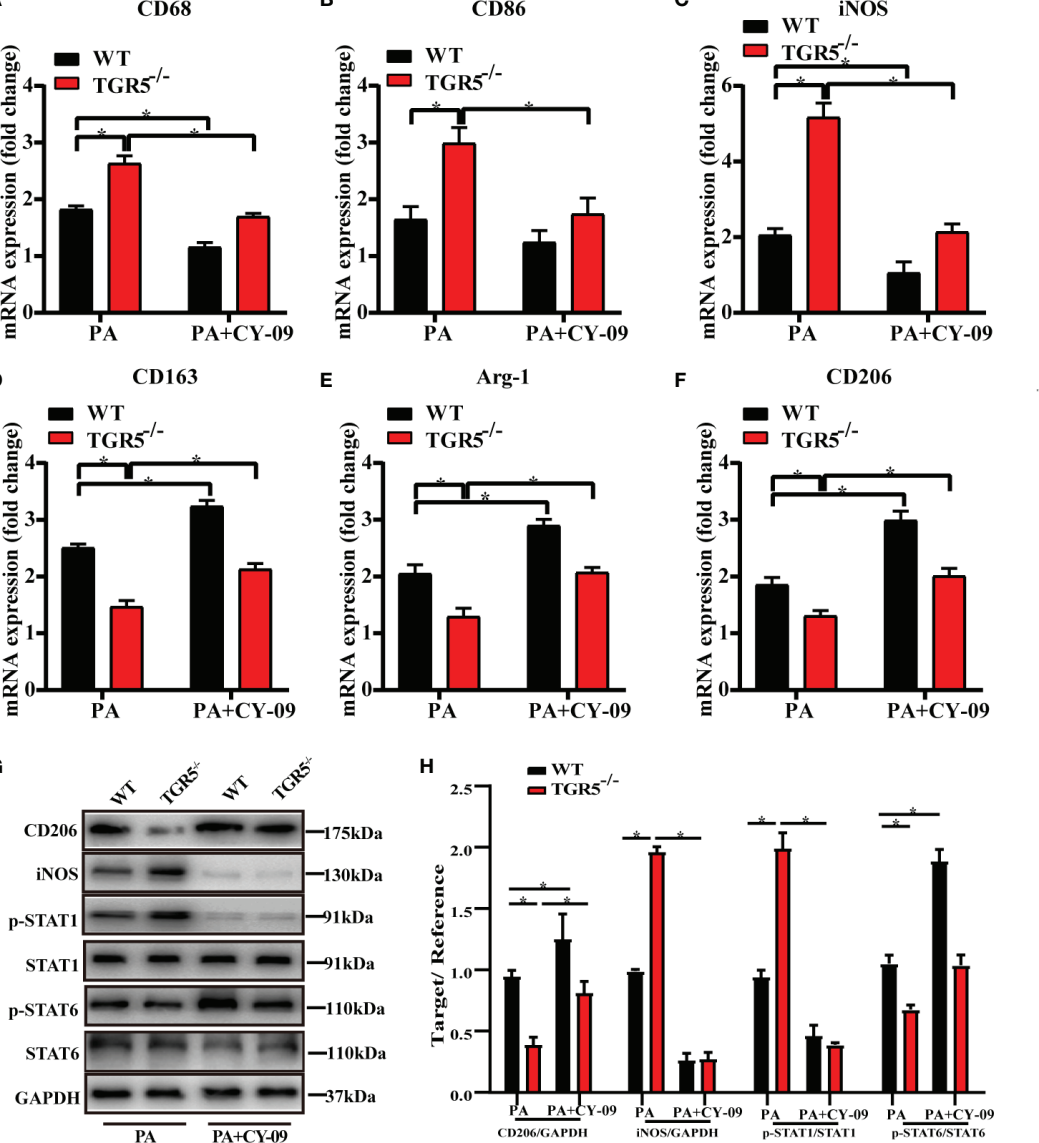
Inhibition of the NLRP3 inflammasome promotes macrophages polarization in TGR5^-/-^ mice with NASH. **(A–F)** mRNA levels of M1 markers (CD68, CD86 and iNOS) and M2 markers (CD163, Arg-1 and CD206) were analyzed by real-time qPCR (n = 3/group). **(G)** CD206, iNOS, p-STAT1, STAT1, p-STAT6, STAT6, and GAPDH protein levels were detected by western blotting. **(H)** The relative quantities of CD206, iNOS protein to GAPDH, p-STAT1 protein to STAT1, p-STAT6 protein to STAT6. Data represent mean ± SD; *P < 0.05.

To further determine the critical role of NLRP3 in mediating the regulation of macrophage activation by TGR5, CY-09, an inhibitor of NLRP3, was used to inhibit NLRP3 activation in WT and TGR5^-/-^ BMDMs post PA stimulation. CY-09 effectively inhibited both the gene and protein levels of NLRP3, IL-1β, IL-18 and caspase-1 (**Figures 6A–G**). Furthermore, treatment with PA and CY-09 resulted in significantly lower levels of TNF-α and IL-6 but higher levels of IL-4 and IL-10 (**Figures 6H, I**), as well as decreased M1 markers, and increased M2 markers (**Figures 7A–F**) both in WT and TGR5^-/-^ BMDMs post-PA stimulation. In addition, NLRP3 inhibition by CY-09 treatment abrogated the role of TGR5 knockout in promoting M1 macrophage activation, as indicated by similar cytokine release and M1/M2 marker expression in WT and TGR5^-/-^ BMDMs (**Figures 7G, H**). These results suggest a critical role for NLRP3 inflammasome activation in mediating M1/M2 macrophage polarization regulated by TGR5.

### TGR5 Inhibits M1 Macrophage Polarization and NLRP3 Inflammasome Activation in Patients With NASH

**Figure 8:**
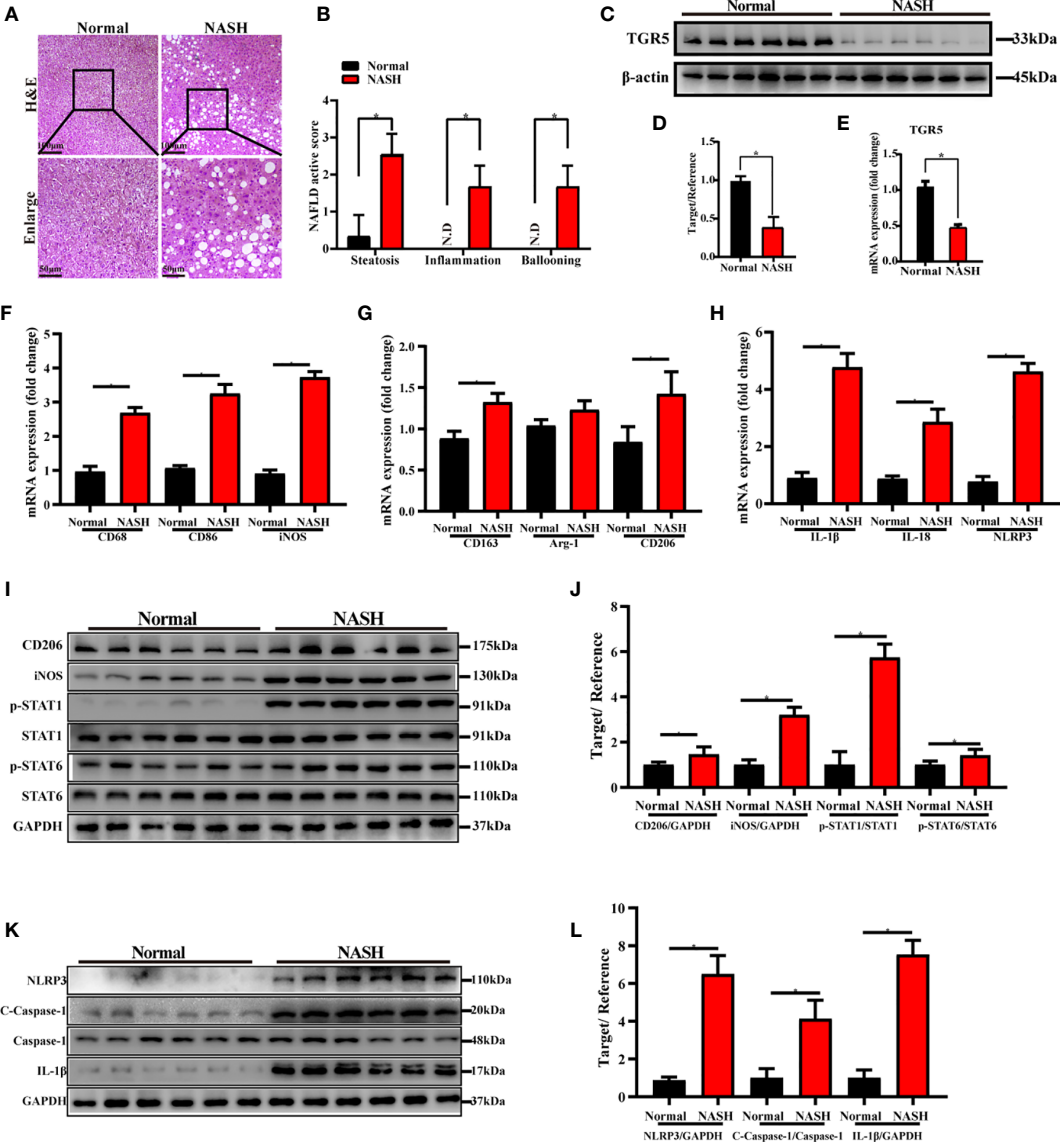
TGR5 inhibits macrophage M1 polarization and NLRP3 inflammasome activation in patients with NASH. **(A)** Human liver specimens were stained with H&E (scale bar, 100 μm and 50 μm, n = 6/group). **(B)** NAFLD activity scores were measured as mentioned in the text. N.D., not detected. **(C)** Expression of TGR5 protein was determined by western blotting. **(D)** Western blots analysis and relative density ratio of protein levels of TGR5. **(E)** Expression of TGR5 mRNA was measured by real-time quantitative PCR. **(F–H)** The mRNA levels of M1 markers (CD68, CD86 and iNOS), M2 markers (CD163, Arg-1 and CD206), IL-1β, IL-18 and NLRP3 were analyzed by real-time qPCR. **(I)** CD206, iNOS, p-STAT1, STAT1, p-STAT6, STAT6, and GAPDH protein levels were detected by western blotting. **(J)** The relative quantities of CD206, iNOS protein to GAPDH, p-STAT1 protein to STAT1, p-STAT6 protein to STAT6. **(K)** Protein levels of NLRP3, c-caspase-1, caspase-1, IL-1β and GAPDH were detected by western blotting in human liver. **(L)** Western blots analysis and relative density ratio of protein levels of NLRP3, c-caspase-1 and IL-1β. Data represent mean ± SD; *P < 0.05.

Finally, we evaluated the role of TGR5 in regulating M1 macrophage polarization and NLRP3 inflammasome activation during NASH in humans. Liver specimens were collected from 6 healthy individuals and 6 NASH patients and were stained with H&E. Compared to normal samples, NASH patients exhibited extensive fat deposition and inflammatory cell infiltration in the liver accompanied by macrovesicular steatosis and ballooning degeneration (**Figure 8A**). The NAFLD activity score of NASH patients was significantly higher compared to normal subjects (**Figure 8B**). Moreover, expression of TGR5 mRNA and protein was lower in liver tissues from NASH patients compared to healthy individuals (**Figures 8C–E**). These results suggest that TGR5 is involved in the development of NASH, which decreases TGR5 expression.

Furthermore, we analyzed the expression levels of M1 markers, M2 markers and NLRP3 in human livers with or without NASH. As shown in **Figures 8F–L**, compared with normal subjects, increased gene and protein expression of M1 markers and enhanced activation of NLRP3 signaling pathway were found in NASH patients. Interestingly, significantly increased expression of M2 markers of CD163, CD206 and p-STAT6/STAT6 ratio and slightly increased expression of Arg-1 were found in NASH patients as well.

## Discussion

**Figure 9:**
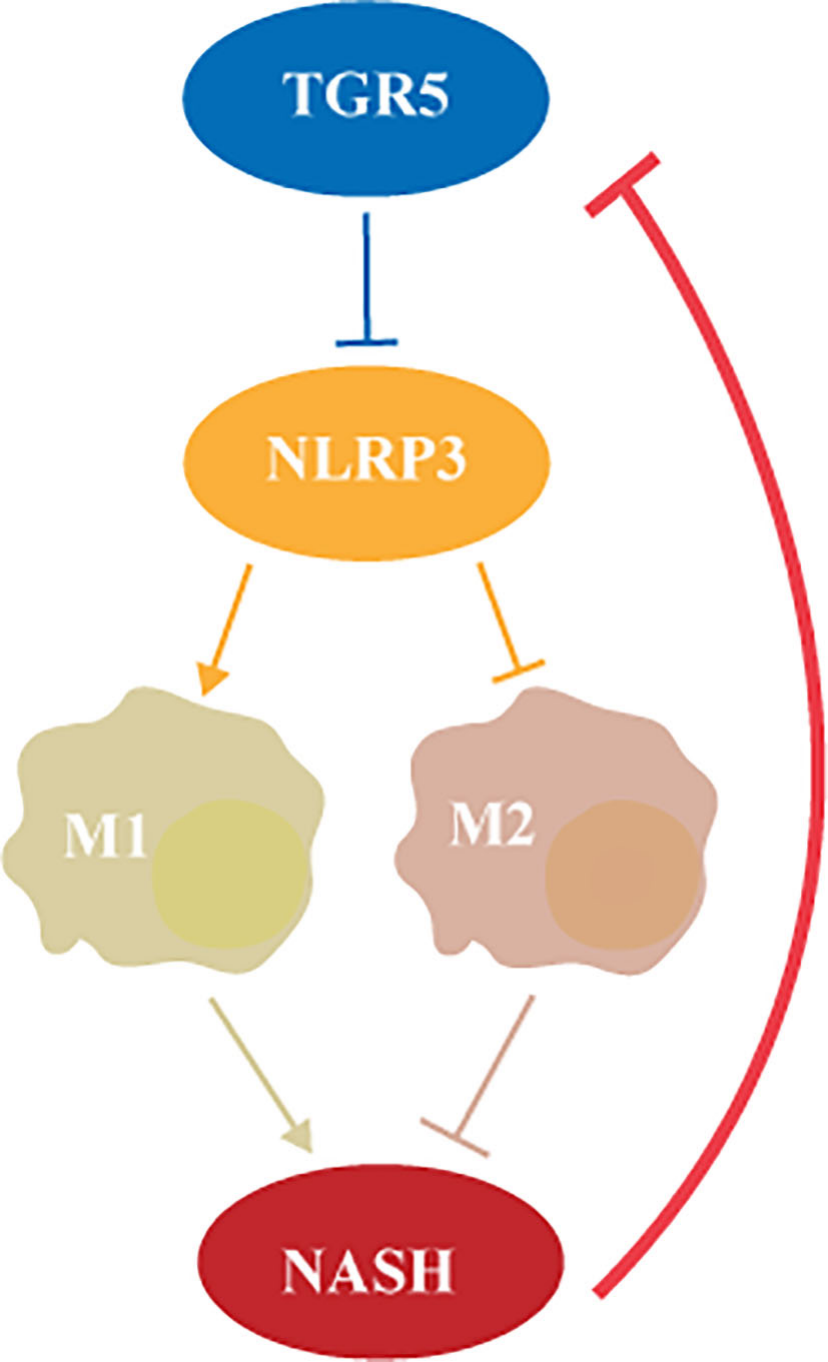
TGR5 regulates macrophage inflammation in nonalcoholic steatohepatitis by modulating NLRP3 inflammasome activation.

In this study, we investigated the role of TGR5 in regulating the activation of macrophages in NASH. Decreased TGR5 expression was found in both murine and human liver samples with NASH. TGR5 deficiency promoted liver steatosis and inflammation by inhibiting M2 macrophage polarization *via* NLRP3 activation. To the best of our knowledge, we demonstrated for the first time that TGR5/NLRP3 signaling modulates M1/M2 macrophage polarization in NASH (**Figure 9**).

NAFLD is a metabolic disease characterized by excessive fat deposition in hepatocytes in addition to alcohol and other definitive factors of liver damage. As a pathological process of NAFLD, nonalcoholic steatohepatitis is histologically characterized by liver biopsy showing lobular inflammation, macrovesicular steatosis, and hepatocyte ballooning (3). Studies have declared that among diagnosed NASH patients, approximately 15 to 50% of patients have severe fibrosis, while 7 to 26% of patients have cirrhosis (28). Recent studies have shown that the development of NASH is closely related to lipid metabolism disorders and oxidative stress (29). In our study, mice fed MCD diet to induce NASH. Among the animal models of NASH, MCD diet is one of the most common easiest methods. It is widely accepted and widely used in recent studies (30). The advantages of the MCD diet are that it replicates NASH histological features observed in human NASH in a relatively shorter feeding time, as compared to the other dietary models. At 3 weeks of MCD feeding, steatohepatitis is well developed, and pericellular and perisinusoidal fibrosis are present at 8–10 weeks (31). However, regarding the complexities of pathogenesis of human NASH, two of more NASH models applied in one study would be much better to confirm the role of TGR5 in regulating NASH development. Both the male and female mice could develop NASH. However, it has reported that global incidence of NASH is higher in men than women. Indeed, both sex and age have been reported as key factors that influence the incidence of NAFLD (32). Thus, to avoid the effect of sex and age on our experiments, only male mice of 6–8 weeks were used in our study. It will be much better to include both the male and female mice in further studies, which would provide more solid evidence to support our findings and reflect the human patient population.

TGR5 is a bile acid receptor widely expressed in multiple cells in the liver, including Kupffer cells and sinusoidal endothelial cells. TGR5 signaling regulates glucose homeostasis and energy expenditure, which is involved in multiple metabolic pathways *in vivo*. Previous studies have indicated that activating TGR5 improves obesity and insulin resistance, which may be related to increased secretion of glucagon-like peptide-1 (GLP-1) in the intestine (33). Here, we observed a decrease in TGR5 expression in humans and mice with NASH. It has been reported that mRNA and protein expression of TGR5 are decreased in NASH mice (34). Furthermore, treatment of HFD mice and db/db mice with phenolic blueberry extract induces expansion of an LCA-producing microbiome, increases TGR5 expression, and improves metabolic disorders in mice (35). Furthermore, the precise mechanism of TGR5 inhibition/deficiency in NASH is still not clear. Studies have shown that TGR5 could be activated by bile acids and lithocholic acid. In NASH, the expression of TGR5 may be inhibited by the dysregulation of bile acids metabolism and decrease of lithocholic acid (36). In addition, energy metabolism disorders and inflammatory responses may also contribute to TGR5 reduction during NASH (37).

Macrophages play an important role in innate immunity by quickly recognizing and clearing substances considered foreign and harmful and exert rigorous regulation of inflammation (38, 39). It is believed that liver damage may be due to macrophages not being able to correctly recognize and eliminate dangerous molecules or a failure to prevent inflammation (40). In addition, macrophages in adipose tissue also affect inflammation by secreting cytokines and adipokines (41). In the NASH model of mice fed a lipotoxic high-fat and high-cholesterol (HFHC) diet, infiltration of F4/80^+^ macrophages and activation of KCs were observed, and expression of proinflammatory cytokines was increased (42). These inflammatory cytokines further magnify liver inflammation and damage hepatocyte function, leading to cell death. Some studies have shown that liver inflammation can impair insulin sensitivity, and selective depletion or inhibition of KCs by clodronate liposomes improves fasting hyperglycemia and insulin resistance in mice fed a high-fat diet (HFD) (43).

Earlier studies have demonstrated that TGR5 is highly expressed in macrophages, and the secretion of these cells and their proinflammatory cytokines is closely related to the progression of NASH, suggesting that TGR5 may be involved in the regulation of macrophages in NASH (40). Our previous research has shown that TGR5 attenuates hepatic ischemia and reperfusion injury by suppressing the TLR4-NF-κB pathway, which may play a key role in reducing related inflammation (44). Actually, in the inflammatory response, stimulation of TGR5 in macrophages reduces inflammation by inhibiting the transcriptional activation of NF- κB, leading to decreased expression of inflammatory cytokines, such as TNF-α, IL-6 and monocyte chemoattractant protein-1 (MCP-1), as well as inhibition of phagocytosis and immune cells migration (45). Additionally, the study revealed that in a model of adipose tissue inflammation and diabetes, TGR5 deficiency in macrophages resulted in increased chemokine expression and migration, which may be related to the AKT-mTOR-LIP axis (46). In this study, we demonstrated that a lack of TGR5 in macrophages increased the secretion of proinflammatory cytokines.

As studies have shown, M1 and M2 are two activation states of macrophages that are essential for maintaining homeostasis through proinflammatory and anti-inflammatory activities, respectively. STAT1 and IRF5 control the M1 macrophage phenotype, while polarization to M2 macrophages is regulated by STAT6 and IRF4 (47). Spermine, a modulator of cell growth and differentiation, has been demonstrated to promote M2 polarization of macrophages in thioacetamide (TAA)-induced liver injury *via* upregulation of ATG5-dependent autophagy (48). Furthermore, Zhou et al. found that peroxisome proliferator-activated receptor-γ (PPARγ)/STAT3 signaling promoted M2 macrophage activation in murine liver ischemia and reperfusion injury. In these studies, the reduction in inflammation was parallel to M2 macrophage polarization. We also illustrated that deficiency of TGR5 promotes M1 polarization of macrophages. These findings suggest that activation of TGR5 in macrophages alleviates levels of proinflammatory cytokines in NASH, possibly by promoting the M2 macrophage polarization.

NLRP3 belongs to the NLR protein family and is involved in immunity and human diseases (49). With activation of the NLRP3 inflammasome, procaspase-1 is cleaved into caspase-1, and pro-IL-1β and pro-IL-18 are converted into mature forms followed by IL-1β and IL-18 secretion (50). Caspase-1 is involved in the inflammatory response and induces proinflammatory cell death (51). In addition, as a proinflammatory mediator, IL-1β participates in a variety of immune responses, including recruiting cells and regulating adaptive immune cells, while IL-18 has a vital effect on the production of interferon-γ (IFN-γ) (52). Studies have shown that NLRP3 plays a role in metabolism. In NLRP3-deficient mice fed a high-fat diet, the mice appear to be sensitive to insulin (53). In addition, due to reduced NLRP3 levels, type 2 diabetic patients have increased insulin resistance after caloric restriction and exercise (54). We previously examined the role of diabetes in liver ischemia and reperfusion injury. Results showed that diabetes promoted activation of the NLRP3 inflammasome regulated by oxidative stress to induce hepatocyte pyroptosis (55). Interestingly, the development of NASH is closely related to metabolic disorders, suggesting that NLRP3 may be involved in NASH. Some studies have suggested that excessive lipid accumulation in NASH is released when hepatocytes die, which activates macrophages to express the NLRP3 inflammasome and caspase-1 at high levels (20). Moreover, in diet-induced NASH, mice with TLR2 deficiency exhibited reduced NLRP3 inflammasome activation and decreased inflammation and fibrosis, and after inhibiting NLRP3, fibrosis was significantly attenuated (56).

Additionally, activation of TGR5 signaling regulates energy expenditure and metabolic homeostasis in the body, indicating that TGR5 may regulate NLRP3 inflammasome activation and macrophage-regulated inflammation. In a recent study, Guo et al. found that activation of bile acids and TGR5 inhibits activation of the NLRP3 inflammasome *via* the TGR5-cAMP-PKA axis, endogenously constraining NLRP3 inflammasome-related inflammation (57). Some studies have shown that liver ischemia and reperfusion injury triggers activation of the NLRP3 inflammasome in macrophages by STING signaling (58). Our data indicated that in a mouse model of NASH with deletion of TGR5, the NLRP3 inflammasome was significantly increased, suggesting that TGR5 attenuated inflammation in NASH by inhibiting activation of NLPR3. Although there is no direct evidence that NLRP3 is related to macrophage polarization, our data may indirectly indicate this conjecture. The present study suggests that M1 macrophages secreted IL-1β and IL-18 rather than M2 macrophages in TGR5^-/-^ mice with NASH, and inhibition of the NLRP3 inflammasome is consistent with M2 polarization.

In summary, there has been a worldwide increase in the prevalence of NASH, but no effective treatment is available (32). Therefore, it is of great significance to study the molecular regulatory mechanisms of the pathogenesis of NASH. This study demonstrates that TGR5 reduces macrophage-related inflammation and promotes macrophage M2 polarization in NASH by inhibiting NLRP3 activation, providing a theoretical basis for targeting TGR5/NLRP3 signaling for NASH treatment.

## Data Availability Statement

The datasets presented in this study can be found in online repositories. The names of the repository/repositories and accession number(s) can be found in the article/**Supplementary Material**.

## Ethics Statement

The studies involving human participants were reviewed and approved by The Institutional Review Board of the First Affiliated Hospital of Nanjing Medical University. The patients/participants provided their written informed consent to participate in this study. The animal study was reviewed and approved by The Institutional Animal Care and Use Committee of Nanjing Medical University.

## Author Contributions

YS, WS, and LZ designed the experiments, performed the experiments, analyzed the data, and wrote the manuscript. YS, WS, CS, JZ, PW, HW, XS, SW, and QW performed the experiments and interpreted the data. JA, KS, and HZ provided research materials. LL, HZ, YY, and RP designed the overall concept, analyzed the data, and wrote the manuscript. All authors contributed to the article and approved the submitted version.

## Funding

This work was supported by grants from National Nature Science Foundation of China (82071798, 81600450, 81570565, 81971495, 81571564, 91442117, 81870448, 31930020), CAMS Innovation Fund for Medical Sciences (No.2019-I2M-5-035), the National Science Foundation of Jiangsu Province (BK20191490, BRA2017533), Six Talent Peaks Project in Jiangsu Province (No. 2018‐WSN‐011), Jiangsu Science and Technology Association Young Science and Technology Talents Lifting Project (No. DG000D4007), the Foundation of Jiangsu Collaborative Innovation Center of Biomedical Functional Materials.

## Conflict of Interest

The authors declare that the research was conducted in the absence of any commercial or financial relationships that could be construed as a potential conflict of interest.
