# Supplementary material for: TGR5 Regulates Macrophage Inflammation in Nonalcoholic Steatohepatitis by Modulating NLRP3 Inflammasome Activation
Source: Front Immunol. 2021 Feb 22;11:609060. doi: 10.3389/fimmu.2020.609060 (PMC7937818; doi:10.3389/fimmu.2020.609060)
Supplement: Supplementary file 1 [file DataSheet_1.docx]

Supplementary Material

# Supplementary Tables

**Supplementary Table 1.** Information about humans

|  | Healthy humans | | | | | | NASH patients | | | | | |
| --- | --- | --- | --- | --- | --- | --- | --- | --- | --- | --- | --- | --- |
| Age | 42 | 43 | 56 | 55 | 48 | 58 | 55 | 48 | 45 | 44 | 52 | 50 |
| Gender | man | man | man | man | woman | man | man | man | man | woman | man | man |
| Steatosis | 0 | 0 | 0 | 0 | 1 | 1 | 3 | 3 | 2 | 3 | 2 | 2 |
| Inflammation | 0 | 0 | 0 | 0 | 0 | 0 | 2 | 2 | 2 | 1 | 1 | 2 |
| Ballooning | 0 | 0 | 0 | 0 | 0 | 0 | 2 | 1 | 1 | 2 | 2 | 2 |
| NAFLD active score | 0 | 0 | 0 | 0 | 1 | 1 | 7 | 6 | 5 | 6 | 5 | 6 |

The severity of NASH is consistent with the score.

**Supplementary Table 2.** Composition of diets

|  | NCD (normal chow diet) | MCD (methionine and choline-deficient diet) |
| --- | --- | --- |
| Total calorie (kcal/g) | 3.41 | 3.91 |
| Carbohydrates (%) | 62.10 | 58.20 |
| Protein (%) | 24.60 | 18.30 |
| Fat (%) | 13.20 | 23.50 |
| Methionine (kcal%) | 0.31 | 0.00 |
